# A method for cancer elemental risk assessments in hookah: An example in two common types of traditional and flavored tobaccos in Iran

**DOI:** 10.1016/j.mex.2023.102431

**Published:** 2023-10-10

**Authors:** Lida Nikbakhtan, Jalal Hassan, Ali Pourshaban-Shahrestani, Seyyed Hamid Ahmadi, Mohammadreza Manafi, Leila Torkian

**Affiliations:** a Department of Chemistry, South Tehran Branch, Islamic Azad University, Tehran, Iran; b Division of Toxicology, Department of Comparative Bioscience, Faculty of Veterinary Medicine, University of Tehran, Tehran, Iran; c Faculty of Clean Technologies, Chemistry and Chemical Engineering Research Center of Iran, Tehran, Iran; d Research center of Modeling and Optimization in Science and Engineering, Islamic Azad University, South Tehran Branch, Tehran, Iran

**Keywords:** Hookah, Flavored tobacco, Traditional tobacco, Elemental analysis, Heavy metals, Modified AOAC

## Abstract

Aim: This study aimed to compare the elemental composition of traditional and flavored hookah tobacco, with a focus on heavy metals. Methods: We used inductively coupled plasma mass spectrometry (ICP-MS) to analyze the concentrations of 29 elements in the raw tobacco, tobacco ash, hookah water after smoking, and tobacco smoke. Results: The results showed that the traditional tobacco had significantly higher metal concentrations than the flavored tobacco in all samples. Most of the toxic metals (more than 98 %) remained in the smoke of both types of tobacco. The tobacco and hookah smoke contained high levels of harmful metals that can pose health risks to hookah users.

• ICP-MS provides a comprehensive analysis of multiple elements simultaneously and it allows for precise quantification of metal concentrations in different samples.
• ICP-MS requires specialized equipment and trained personnel and it may not detect elements present in extremely low concentrations.

## Graphical absract

**Figure:**
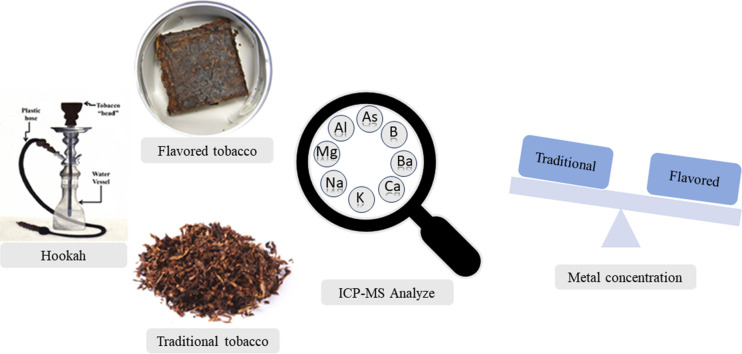

Specifications table

**Table:** 

Subject area:	Environmental Science
More specific subject area:	Sample preparation
Name of your method:	Modified AOAC
Name and reference of original method:	AOAC 2007.01
Resource availability:	It is not applicable

## Method details

 

## Introduction

Smoking is a major health problem in the modern world, and it causes chronic and severe addiction and health consequences. One form of tobacco use is hookah (a type of water pipe) smoking, which has different names in different regions. Most hookahs use a hot wood charcoal to burn the tobacco and produce smoke, which has become very popular among young people in recent years [1], [2], [3], [4]. In Iran, there are two categories of tobacco used: traditional and flavored. Traditional tobaccos are usually named after the cities where they are grown (Khansar, Borazjan, Kashan). The flavored tobaccos come in a wide range of aromas, such as apple, banana, berry, cherry, chocolate, coconut, coffee, cola, grape, kiwi, lemon, licorice, mango, mint, orange, peach, pineapple, rose, strawberry, fruity, vanilla, and watermelon [5,6].

Iranian medical professionals are seriously concerned about the health of their young compatriots who smoke hookah. Flavored products are sold in colorful packages that often do not have health warnings and are aimed at younger consumers. The flavor of hookah tobacco (known as Maassel) is often from molasses, fruit pulp or honey, which makes it have a sweeter smell and taste [7]. In a typical hookah lounge, customers can buy flavored tobacco and rent a pipe for smoking. Before the introduction of Maassel, most water pipe smokers around the world used a type of raw tobacco that was powdered, mixed with water, crushed, and shaped before use. This process usually produced a strong and unpleasant smoke, unlike the smooth and fragrant smoke generated by Maassel [8].

The composition of these products is variable and not standardized. Maassel currently contains about 30 % tobacco and up to 70 % honey or molasses/sugar cane, as well as glycerol and flavoring agents. To make traditional tobacco, chopped tobacco leaves are soaked in water for a few hours to wash off excess nicotine. The levels of this alkaloid vary in different products. Low levels of metals in natural ranges are essential for human health, but high levels due to chronic and cumulative exposure can cause harm to human health [9,10].

Tobacco used in water pipe smoking is an evidenced cause of toxic heavy metals. It has been found that metal contents in tobacco are very different depending upon various parameters, including soil type and pH genotype, the usage of metal pesticides, fertilizers, and so on. Because metals are easily absorbed by the tobacco plant through the soil, their amount varies in each country depending on the cultivation and processing [9], [10], [11]. However, there is limited and inconsistent information on the products derived from tobacco, such as Maassel, which are widely used in shisha with increasing popularity. The levels of elements found in tobacco and their risk assessment are important because they can potentially interact with the main metals. Moreover, exposure to smoking can increase the body's levels of metals and disrupt the metal balance, leading to possible health problems. Therefore, it is necessary to measure the minerals in these substances to evaluate their health effects [12,13]. In this study, we analyzed the concentrations of 29 elements (Al, As, B, Ba, Ca, Cd, Ce, Co, Cr, Cu, Fe, Hg, K, Mg, Mn, Mo, Na, Ni, P, Pb, S, Sb, Se, Si, Sn, Sr, Ti, V, and Zn) in the raw tobacco, tobacco ash, hookah water after smoking and tobacco smoke. We selected the tobacco samples from two common types of Iranian traditional (Khansar and Borazjan) and Maassel (Apple, Blueberry, and Orange) tobaccos. We also performed statistical analysis of the element amounts in different conditions (raw, ash, water, and smoke) for different tobaccos to find the significant differences among the data.

## Materials and methods

We obtained five kinds of water pipe tobaccos that are commonly used in Iran from local shops. We used three types of tobaccos with flavors (Apple, Orange, and Blueberry) and two types of tobaccos with traditional names (Borazjan and Khansar) for this study. The chemicals, HNO3 70 %, and H2O2 30 %, were purchased from Sigma-Aldrich.

### Sample preparation

To determine the ash content, 5.0 g of sample was heated at 600 °C for two hours. For the metal analysis in tobacco and ash, 0.50 g of each sample (homogenized tobacco and its ash) was weighed and transferred to a digestion vessel separately. A reagent blank was prepared by adding Milli-Q™ water instead of the sample for each digestion batch (10 vessels) to monitor the background concentration of the elements. Each digestion vessel was added with 5 mL of concentrated HNO3 (70 %) and 2 mL of H2O2 30 % solution. The samples were left for approximately 1 h (pre-digestion) then sealed in the vessels and digested in the water bath at 90 °C. After cooling, the upper layer was filtered through a paper filter and transferred into acid-washed polypropylene tubes. The samples were diluted to 25 mL with Milli-Q™ water and stored at 4–6 °C in a dark medium until the analysis was performed. The reagent blanks were prepared under the same conditions. The water from the hookah during tobacco burning was transferred into acid-washed polypropylene tubes and analyzed three times.

### The analysis of the tobacco's metal content

The Agilent 7900 ICP-MS with the octupole reaction system (ORS) collision/reaction cell (CRC) was used for the analysis of metal amounts in the digested samples. The ORS was operated in helium collision mode to remove all matrix-based polyatomic interferences. The technique covers the entire essential validation parameters concerning assessments on ICP-MS, including operating conditions, instrument multi-element calibration standards, sensitivity check, and reagent blank. all elements were measured using a single set of operating conditions without switching cell gas modes. The sample introduction system consisted of a Micro Mist glass concentric nebulizer and a quartz double-pass Scott-type spray chamber maintained at a temperature of 4 °C. A standard quartz torch with 2.5 mm internal diameter (ID) injector was used. auto-tuned using the 7900 Mass Hunter software. The instrument was equipped with an Agilent I-AS integrated autosampler. The instrumental parameters are given in Table 1. Ultra-pure water obtained from a Milli-Q purification device (Millipore Co., Bedford, MA, USA). Standard labware and glassware were acid washed and rinsed with Ultra-pure water. Multi-elemental standard solution for ICP-OES contains elements of Al, As, Ba, Bi, Ca, Cd, Ce, Hg, Mg, Na, P, Pb, Se, Sr, Tb, (10 ppm, 26XSM80B.5 L) and Multi-elemental standard solution for ICP-OES contains elements of B, Co, Cr, Cu, Fe, K, Li, Mn, Mo, Ni, Sb, Si, Sn, Ti, V, Zn, (10 ppm, 26XSM90C.5 L), was obtained from MBH, (London, England). The mixed standard solution was obtained by further diluted to desired concentration daily prior to use. The figures of merit for element are given in Table 2.

**Table 1:** Instrumental and operating conditions for ICP-MS measurements.

Instrument parameter	Value
Power (KW)	1.5
Plasma flow rate (L min^−1^)	15
Auxiliary flow rate (L min^−1^)	1.5
Nebulizer flow rate (L min^−1^)	0.7
Helium collision gas (mL min^−1^)	4.2
Point/mass	1
Integration time/point (s)	0.3
Instrument stabilization delay (s)	30
Replicate	3

**Table 2:** Analytical figures of merit of the method.

Elements	Calibration equation	Linear range (µg L^−1^)	Correlation coefficient	LOD (µg L^−1^)	%RSD
Al	*I* = 16,963 *X* + 52,785	1–1000	0.999	0.01	10.3
As	*I* = 3457 *X* + 3966	1–1000	0.999	0.007	6.2
B	*I* = 1389 *X* + 2751	1–1000	0.999	0.2	6.2
Ba	*I* = 23,780 *X* + 22,040	1–1000	0.999	0.01	5.4
Ca	*I* = 458 *X* + 3371	1–1000	0.991	0.1	5.2
Cd	*I* = 2657 *X* + 1212	1–1000	0.999	0.01	8. 5
Ce	*I* = 30,425 *X* + 17,483	1–1000	0.999	0.01	2.5
Co	*I* = 27,048 *X* + 21,859	1–1000	0.998	0.1	4.8
Cr	*I* = 23,326 *X* + 39,693	1–1000	0.999	0.1	11.9
Cu	*I* = 12,201 *X* + 8998	1–1000	0.992	0.01	5.6
Fe	*I* = 720 *X* + 34,427	10–1000	0.999	4.1	6.6
Hg	*I* = 352 *X* + 372	1–1000	0.997	0.03	5.6
K	*I* = 52,393 *X* + 2,000,000	1–1000	0.993	0.5	5.4
Mg	*I* = 17,032 *X* + 30,944	1–1000	0.999	0.01	3.4
Mn	*I* = 32,697 *X* + 28,650	1–1000	0.998	0.4	4.6
Mo	*I* = 7392 *X* + 16,554	1–1000	0.998	0.01	3.9
Na	*I* = 35,901 *X* + 31,725	1–1000	0.991	0.01	3.7
Ni	*I* = 5843 *X* + 4775	1–1000	0.999	0.02	5.1
P	*I* = 1238X+113,344	50–5000	0.998	43	8.4
Pb	*I* = 8688 *X* + 1752	1–1000	0.999	0.02	9.1
S	*I* = 11,231 *X* + 527,851	20–2000	0.996	8.1	5.6
Sb	*I* = 8692 *X* + 366	1–1000	0.999	0.02	6.6
Se	*I* = 269 *X* + 403	1–1000	0.996	0.4	9.3
Si	*I* = 12,169 *X* + 369,226	20–2000	0.993	6.1	5.4
Sn	*I* = 8218 *X* + 9982	1–1000	0.998	0.02	3.4
Sr	*I* = 33,156 *X* + 40,568	1–1000	0.999	0.01	4.5
Ti	*I* = 11,942 *X* + 362	1–1000	0.999	0.02	9.9
V	*I* = 33,246 *X* + 28,862	1–1000	0.999	0.02	5.7
Zn	*I* = 3352 *X* + 15,555	1–1000	0.9964	0.1	5.2

### Statistical analysis

The plots are the result of processing data in Microsoft Excel 2019. Since three observations are too small to check out the statistical presumptions like normality, equality of variances, and so on. We are forced to use non-parametric tests. As water is different from the two other groups (confirmed by all plots and all tests even with P-value = 0.05), we concentrate on the comparison of raw and ash ingredients using a paired analysis. In consequence, the data was analyzed in SPSS 21 in terms of the Friedman test (P-value 0.10).

## Results and discussion

### The metals in raw tobaccos

**Fig. 1:**
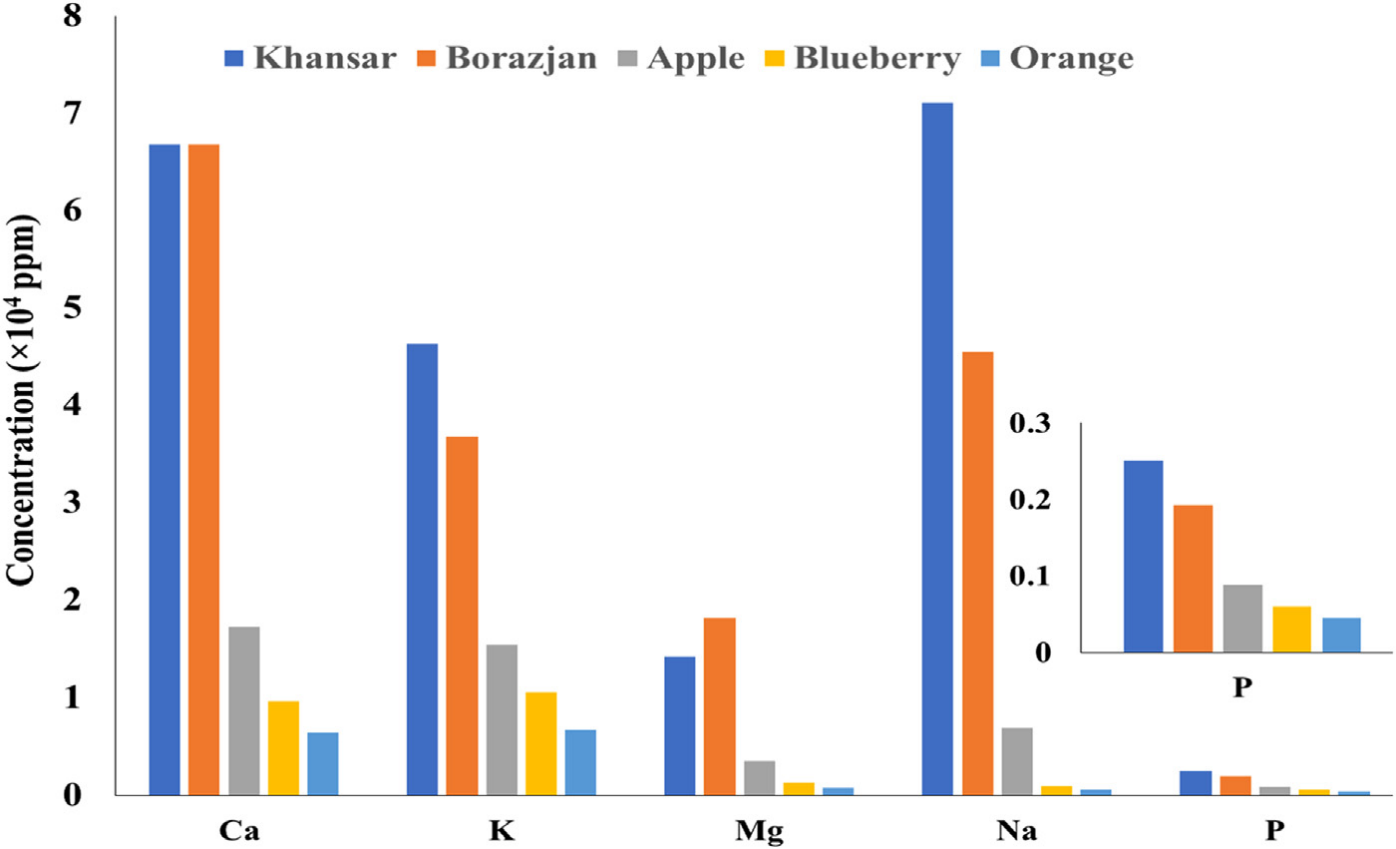
The portion of the main metals in the raw tobaccos.

The minerals determination in raw tobacco was performed. Fig. 1 shows the portion of the main metals in the 5 samples of the raw tobaccos. These five metals, Ca, K, Na, Mg, and P make up respectively 97.2 %, 99.6 %, 95.0 %, 96.8 %, 97.2 % of the total concentration of Apple, Blueberry, Orange, Khansar, and Borazjan tobaccos.

Generally, 29 metals were detected in two types tobacco Maassel and traditional. Results showed that the total content of metals (mg.kg^−1^) was more abundant in traditional tobacco than Maassel. Table 3 shows the concentrations of Al, As, B, Ba, Ca, Cd, Ce, Co, Cr, Cu, Fe, Hg, K, Mg, Mn, Mo, Na, Ni, P, Pb, S, Sb, Se, Si, Sn, Sr, Ti, V, and Zn for each raw tobacco sample. As shown in this table calcium and potassium contents of Maassel (Apple, Blueberry, and Orange) are significantly higher than those of traditional (Borazjan and Khansar). This implies that Maassel tobaccos may have higher levels of additives or flavorings that contain these elements, which may affect the pH and buffering capacity of the smoke and water. Previous studies have suggested that higher pH and buffering capacity may increase the absorption of nicotine and other harmful substances by the mucous membranes of the mouth and respiratory tract Therefore, Maassel tobaccos may pose greater risks of nicotine dependence and oral diseases than traditional tobaccos [14]. However, in the case of sodium and magnesium, traditional tobaccos have higher content than Maassel.

**Table 3:** The concentration of the metals in the raw tobacco's structures.

	Al (ppm)	As (ppb)	B (ppm)	Ba (ppm)	Ca (ppm)	Cd (ppb)	Ce (ppm)	Co (ppm)	Cr (ppm)	Cu (ppm)
Apple	182.3	66	134.2	16.4	17,214	221	1.4	<0.5	<0.5	5.2
Blueberry	197.4	<50	121.3	14.2	9676	231	0.5	0.8	<0.5	3.7
Orange	171.4	3093	116.9	8.4	6418	346	<0.5	0.8	<0.5	2.3
Khansar	1836.2	462	223.4	118.9	66,743	269	3.9	2.7	4.7	12.8
Borazjan	984.6	237	223.6	60.4	66,797	328	2.6	1.2	2.8	12.9
	Fe (ppm)	Hg (ppb)	K (ppm)	Mg (ppm)	Mn (ppm)	Mo (ppm)	Na (ppm)	Ni (ppm)	P (ppm)	Pb (ppm)
Apple	256.4	23	15,422	3503	40.4	0.3	6932.1	2.2	874.2	3.3
Blueberry	78.1	21	10,576	1362	48.6	<0.5	983.1	<0.5	595.6	1.5
Orange	47.8	229	6711	752	42.7	<0.5	544.3	<0.5	448.1	3.8
Khansar	2360.9	72	46,276	14,179	130.7	2.9	71,050.0	8.0	2513.0	9.7
Borazjan	1415.1	47	36,826	18,108	113.7	3.9	45,489.0	4.9	1928.5	5.9
	S (ppm)	Sb (ppm)	Se (ppm)	Si (ppm)	Sn (ppm)	Sr (ppm)	Ti (ppm)	V (ppm)	Zn (ppm)	
Apple	198	<1	1.3	203.5	3.7	161.8	2.4	0.7	16.2	
Blueberry	100	<1	1.9	192.0	1.9	41.9	<1	<0.5	14.6	
Orange	70	<1	4.7	182.1	<1	26.6	<1	<0.5	12.2	
Khansar	683	<1	4.1	243.7	2.3	699.1	25.8	4.2	30.7	
Borazjan	937	<1	3.3	166.8	1.6	924.2	19.2	3.1	29.7	

### The metals in tobacco's ash

**Fig. 2:**
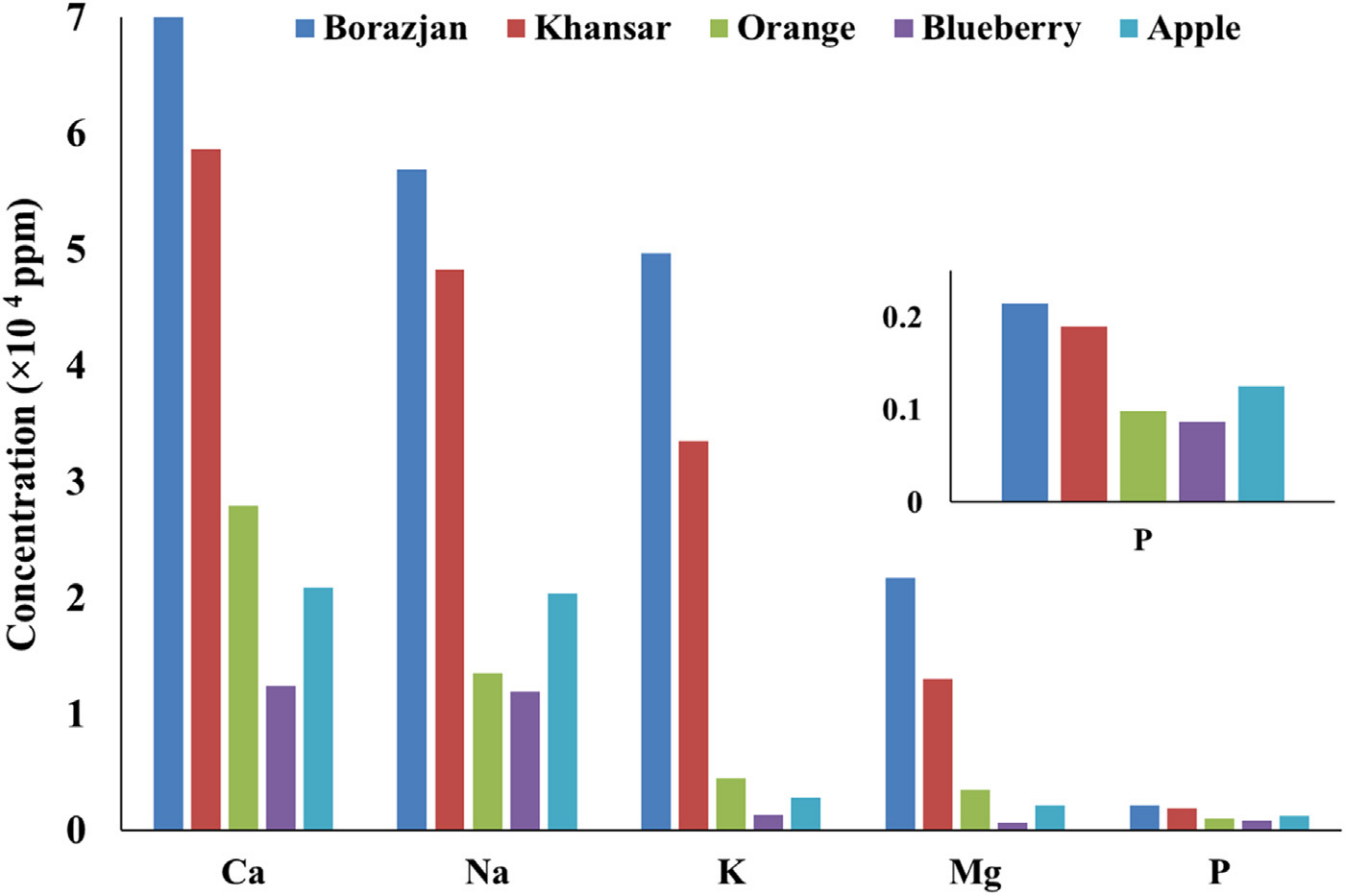
The concentration of the main metals in the tobacco's ash.

The metal content of the tobacco's ash was measured. Fig. 2 shows the concentration of the main metals in the samples of the tobacco's ash. The Ca, K, Na, Mg, and P make up 97.6 %, 96.7 %, 97.3 %, 97.4 %, 97.7 % of the total concentration of Apple, Blueberry, Orange, Khansar, and Borazjan tobaccos. The concentration of the main metals in the Blueberry ash has a significant difference from that of Blueberry raw tobacco, and no significant differences were observed for others. These differences can be related to the different carbon content of the tobacco's chemical structures. The more carbon content of raw tobacco, the more metal concentration in its ash. Table 4 shows the concentrations of Al, As, B, Ba, Ca, Cd, Ce, Co, Cr, Cu, Fe, Hg, K, Mg, Mn, Mo, Na, Ni, P, Pb, S, Sb, Se, Si, Sn, Sr, Ti, V, and Zn for each tobacco ash.

**Table 4:** The concentration of the metals in the tobacco's ash.

	Al (ppm)	As (ppb)	B (ppm)	Ba (ppm)	Ca (ppm)	Cd (ppb)	Ce (ppm)	Co (ppm)	Cr (ppm)	Cu (ppm)
Apple	138.1	2031	126.2	18.3	20,890	406	1.1	1.0	<0.5	15.3
Blueberry	195.0	339	117.5	16.8	12,481	806	<0.5	0.5	<0.5	6.2
Orange	307.1	<50	132.8	21.6	27,930	80	<0.5	0.3	<0.5	9.7
Khansar	1043.1	316	209.6	77.1	58,661	375	1.5	1.7	2.7	9.4
Borazjan	846.4	<50	248.6	56.3	70,561	318	2.5	1.3	2.3	17.5
	Fe (ppm)	Hg (ppb)	K (ppm)	Mg (ppm)	Mn (ppm)	Mo (ppm)	Na (ppm)	Ni (ppm)	P (ppm)	Pb (ppm)
Apple	173.4	<50	20,424	2850	40.4	<0.5	2133.1	8.7	1249.2	6.5
Blueberry	131.0	<50	11,913	1318	55.5	<0.5	700.9	4.4	862.1	2.1
Orange	280.3	126	13,550	3457	24.2	0.6	4493.5	2.8	982.1	6.7
Khansar	1357.3	38	33,543	13,058	114.4	3.0	48,296.0	6.3	1897.2	3.8
Borazjan	1126.5	<50	49,687	21,759	129.3	4.7	56,909.0	5.9	2151.3	3.1
	S (ppm)	Sb (ppm)	Se (ppm)	Si (ppm)	Sn (ppm)	Sr (ppm)	Ti (ppm)	V (ppm)	Zn (ppm)	
Apple	240	<1	<1	256.2	<1	113.3	1.4	<0.5	25.8	
Blueberry	146	<1	<1	172.1	2.2	53.6	<1	<0.5	19.4	
Orange	188	<1	1.4	208.8	1.4	178.8	2.8	0.5	17.2	
Khansar	490	<1	1.0	118.7	1.0	663.1	17.0	3.1	26.0	
Borazjan	752	<1	4.1	127.4	5.7	1260.6	16.6	3.2	42.7	

### The metals in the water after tobacco burning

**Fig. 3:**
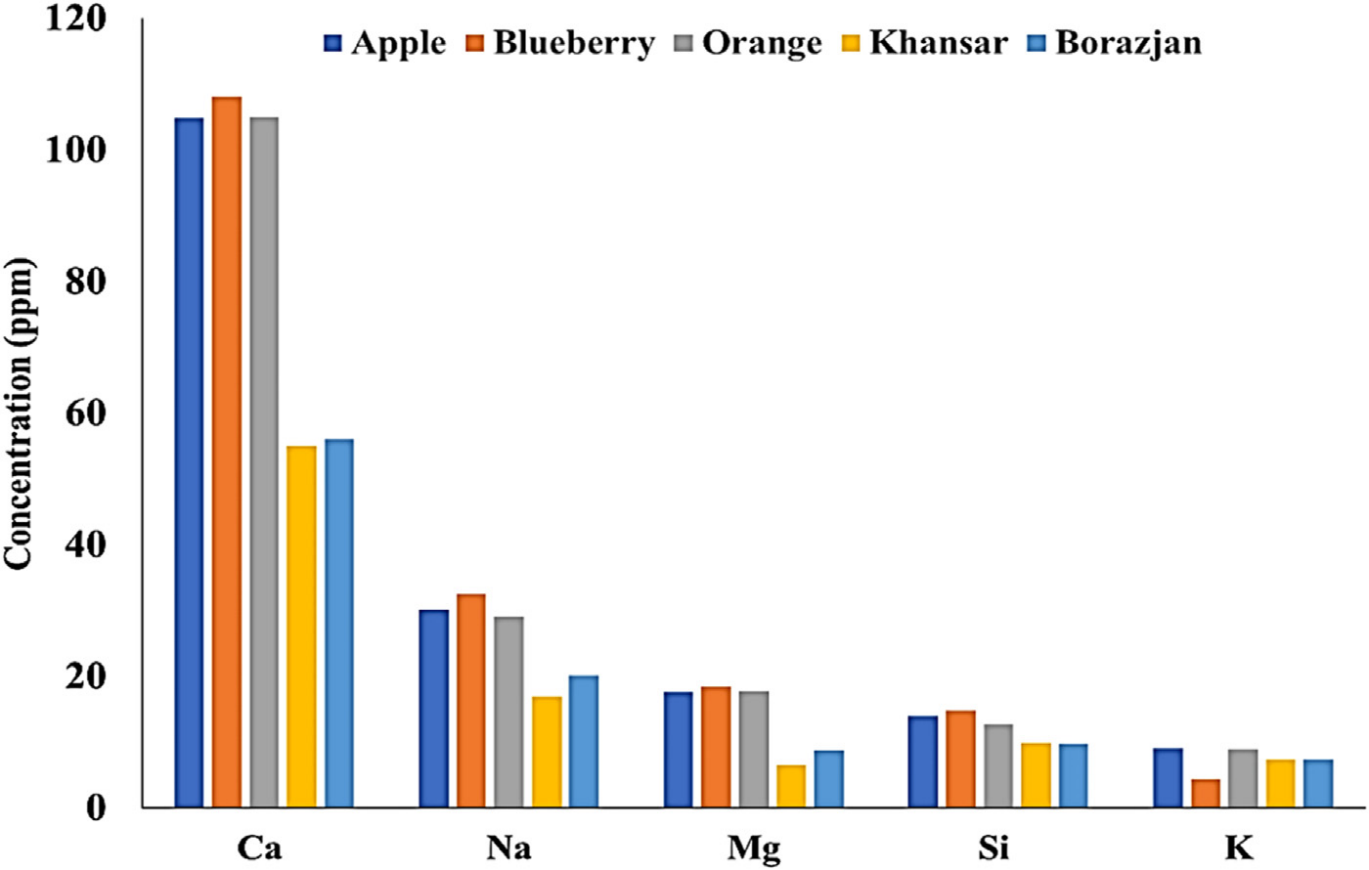
The concentration of the main metals in the water after tobacco burning.

The metals that were dissolved in the water of the water pipe during tobacco burning were detected and measured. The dissolution of the metals in the water is dependent on the nature of the salts of the metals which determines the amount of the salt dissolution in water. Results showed that the concentrations of the elements in the water are significantly lower than raw tobaccos and tobacco's ash. It can be related to the fact that there is a limitation for the elements especially heavy metals to fly through the smoke and enter the water during smoking. Fig. 3 shows the concentration of the main metals in the samples of the tobacco's ash. The Ca, K, Na, Mg, and P make up 98.6 %, 98.3 %, 98.5 %, 98.8 %, 98.9 % of the total concentration of Apple, Blueberry, Orange, Khansar, and Borazjan tobaccos. The very close results are probably due to the fact that the solubility of the salts of these metals in water is very low.

Table 5 shows the concentrations of Al, As, B, Ba, Ca, Cd, Ce, Co, Cr, Cu, Fe, Hg, K, Mg, Mn, Mo, Na, Ni, P, Pb, S, Sb, Se, Si, Sn, Sr, Ti, V, and Zn for each tobacco ash.

**Table 5:** The concentration of the metals the water after tobacco burning.

	Al (ppm)	As (ppb)	B (ppm)	Ba (ppm)	Ca (ppm)	Cd (ppb)	Ce (ppm)	Co (ppm)	Cr (ppm)	Cu (ppm)
Apple	213	6	739	83	105	<5	<10	<10	<10	11
Blueberry	81	<5	687	85	108	<5	<10	<10	<10	26
Orange	66	28	668	87	105	<5	<10	<10	<10	24
Khansar	52	<5	657	22	55	<5	<10	<10	<10	29
Borazjan	36	<5	651	24	56	<5	<10	<10	<10	<20
	Fe (ppm)	Hg (ppb)	K (ppm)	Mg (ppm)	Mn (ppm)	Mo (ppm)	Na (ppm)	Ni (ppm)	P (ppm)	Pb (ppm)
Apple	46	<10	8.96	17.59	42	<20	30.09	<20	416	16
Blueberry	42	<10	4.42	18.49	19	<20	32.56	39.00	332	384
Orange	67	<10	8.86	17.72	10	<20	29.02	<20	36	196
Khansar	<50	<10	7.39	6.55	12	<20	16.91	<20	51	<20
Borazjan	<50	<10	7.40	8.76	15	<20	20.12	<20	67	22
	S (ppm)	Sb (ppm)	Se (ppm)	Si (ppm)	Sn (ppm)	Sr (ppm)	Ti (ppm)	V (ppm)	Zn (ppm)	
Apple	*****	<20	<20	13.90	<20	694	<50	<50	58	
Blueberry	*****	<20	<20	14.77	53	751	<50	<50	217	
Orange	*****	<20	<20	12.69	22	722	<50	<50	72	
Khansar	*****	<20	<20	9.81	<20	303	<50	<50	26	
Borazjan	*****	<20	<20	9.76	<20	339	<50	<50	29	

### The metals in tobaccos smoke

**Fig. 4:**
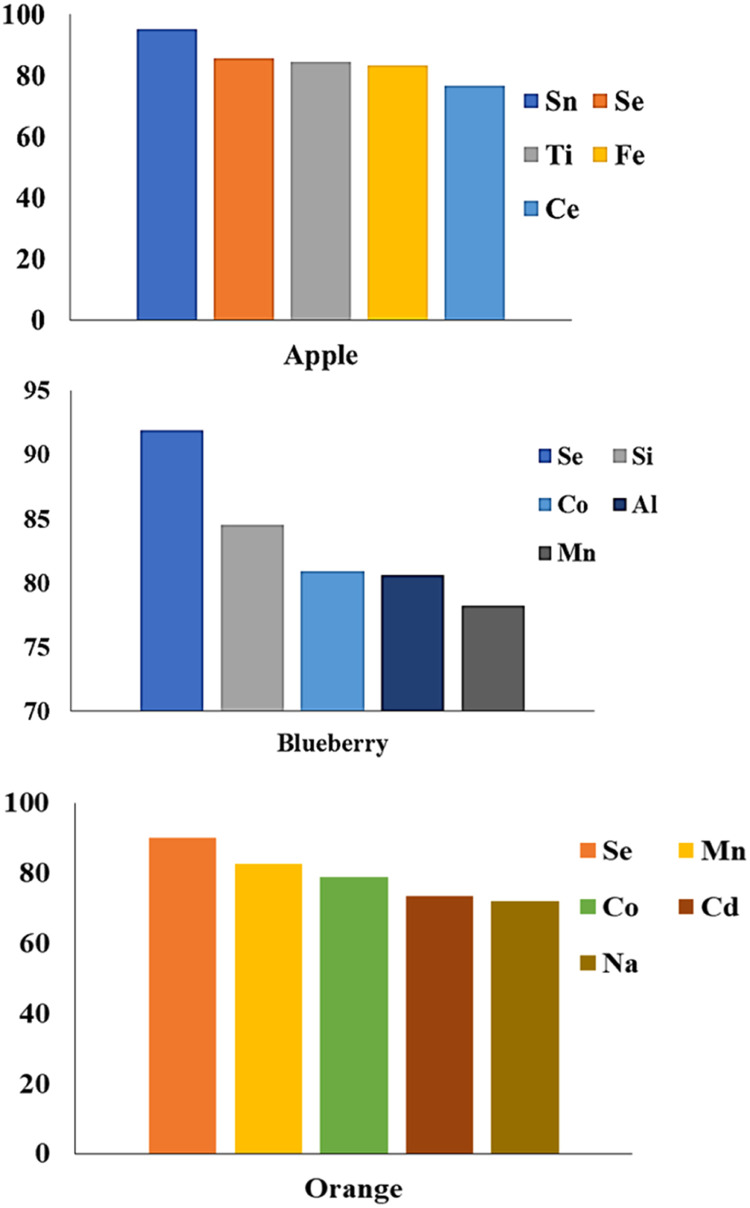
The percentage of remaining main elements in the smoke of Maassel tobaccos.

The percentage of trace elements in tobacco smoke was calculated by subtracting the sum of each in the ash and water from their amounts in the raw tobacco. The results showed that there are between 69.5–93 % of the raw tobacco's metal content in their smoke. This means that these elements can enter the body of the users, and in this regard, there is no significant difference between traditional and flavored tobacco. Fig. 4 shows the percentage of remaining main elements in the smoke of Maassel tobaccos. As shown in the figure, selenium is the only element that is among the main elements in all types of Maassel. Although the main elements in tobacco smoke are different, the presence of such toxic elements in tobacco smoke can cause great harm to the body. For instance, Ni and Cd are known carcinogens that can damage the DNA and cause mutations that lead to cancer [14]. Ni can also cause allergic reactions, asthma, and lung inflammation. Cd can affect the kidneys, bones, and blood pressure. Cr is another carcinogen that can cause lung cancer, especially in workers exposed to chromium compounds. Cr can also cause skin ulcers, allergic dermatitis, and respiratory problems [15]. These elements were found in high concentrations in raw and ash states of tobaccos, indicating that they are not filtered out by water or smoke. Therefore, hookah smokers are exposed to these elements through inhalation or ingestion.

**Fig. 5:**
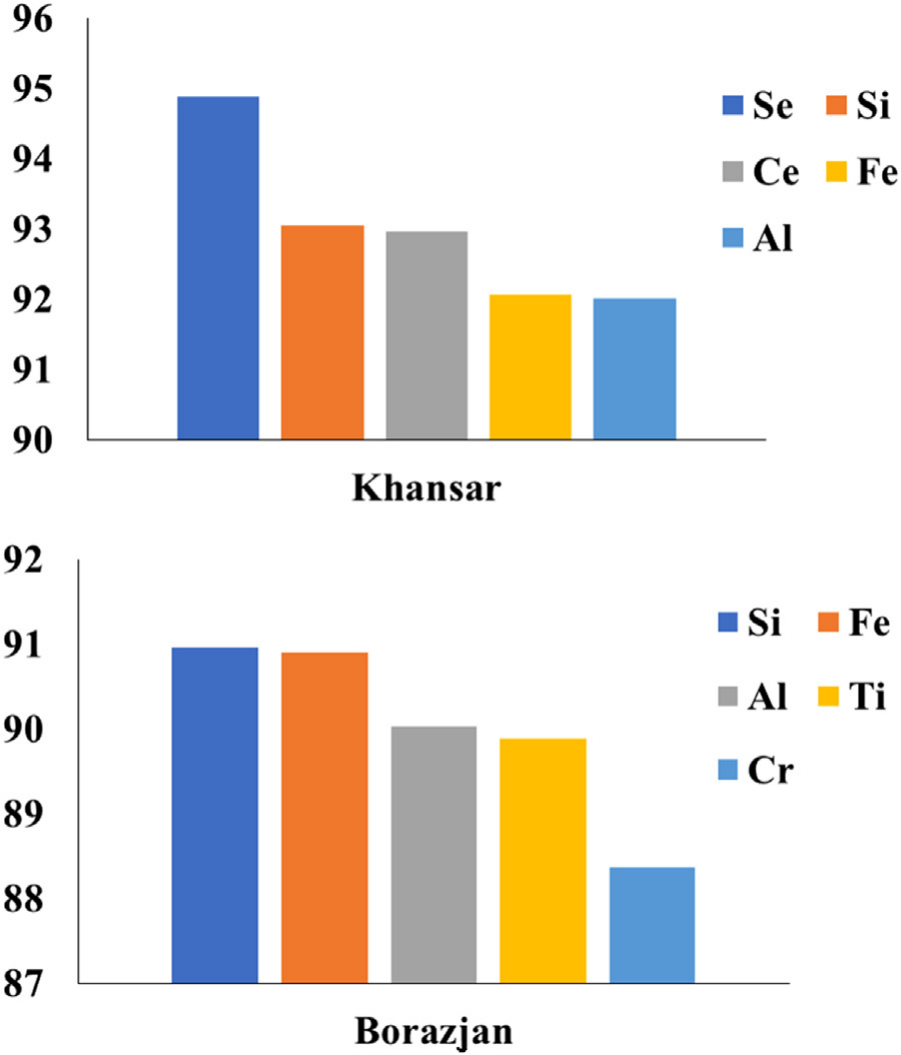
The percentage of remaining main elements in the smoke of traditional tobaccos.

Fig. 5 shows percentage of remaining main elements in the smoke of traditional tobaccos. In the case of Borazjan, generally, the percentage of the remaining metals is lower than Khansar. Although, Cr is one of the main remaining elements, the Borazjan smoke seems to be less toxic.

Table 6 shows the percentage of the remaining elements in Maassel and traditional tobaccos. As shown in the table significant percentages of the elements remained after burning all tobaccos in their smoke. This is a very important result that revealed the ability of hookah to damage the body. It reveals the ability of hookah to damage the body by delivering high levels of toxic elements to the lungs and other organs. Some of these elements are known to cause cancer, such as nickel, cadmium, and chromium. Others can cause allergic reactions, asthma, lung inflammation, kidney damage, bone loss, blood pressure problems, skin ulcers, and respiratory infections.

**Table 6:** The percentage of the remaining elements in Apple, Blueberry, Orange, Khansar, Borazjan tobaccos.

	Apple	Blueberry	Orange	Khansar	Borazjan
**Al**	74.6	80.6	47.7	92.0	90.0
**As**	64.0	21.8	24.5	56.8	57.8
**B**	40.1	44.2	28.5	66.6	67.1
**Ba**	39.2	38.6	34.5	89.8	86.7
**Ca**	29.8	3.5	4.3	67.7	67.8
**Cd**	26.6	11.4	73.4	54.9	67.7
**Ce**	76.6	71.2	58.3	93.0	86.9
**Co**	39.6	80.9	78.9	89.0	81.2
**Cr**	63.3	69.3	58.3	90.6	88.4
**Cu**	17.5	22.9	57.6	74.0	73.8
**Fe**	83.4	68.2	44.3	92.1	90.9
**Hg**	68.4	56.3	54.3	48.3	43.0
**K**	65.9	78.3	34.8	89.0	83.3
**Mg**	46.4	55.0	6.2	84.2	83.0
**Mn**	70.0	78.3	82.6	87.4	86.2
**Mo**	41.4	69.3	52.5	83.5	84.7
**Na**	62.6	74.0	72.0	89.0	82.8
**Ni**	5.7	67.9	65.9	88.3	85.0
**P**	64.2	71.9	38.7	89.5	87.2
**Pb**	21.6	18.7	14.1	80.0	68.1
**S**	37.2	5.8	28.3	79.9	83.5
**Sb**	63.3	69.3	58.3	72.3	74.7
**Se**	85.6	91.9	90.0	94.9	83.5
**Si**	70.9	84.5	67.7	93.0	91.0
**Sn**	95.1	77.2	68.9	90.9	55.6
**Sr**	54.1	56.6	21.0	84.0	82.0
**Ti**	84.4	69.3	70.5	90.7	89.9
**V**	73.5	69.3	60.8	88.3	86.0
**Zn**	38.8	67.5	19.9	82.5	76.9

The results of Friedman test (Pre: Raw Post: Ash Grouping: Traditional vs Maassel) for different elements are provided in Table 7. The table confirms that the concentration of traditional tobaccos is more than Maassel ones. Generally, the ash of traditional has a lower concentration in comparison with the raw material.

**Table 7:** The results of Friedman test for different elements.

Element	P-value	Comparison of type	Comparison of time	Interaction effect
Ca	*	Traditional > Maassel	Ash > Raw	The content of the ash of traditional tobaccos in lower than its raw.
Na	–	Traditional > Maassel		
K	*	Traditional > Maassel	Ash > Raw	The content of the ash of traditional tobaccos equals its raw.
Mg	–	Traditional > Maassel		
P	–	Traditional > Maassel		
Fe	*	Traditional > Maassel	Ash < Raw	The content of the ash of Maassel tobaccos in higher than its raw.
Al	*	Traditional > Maassel	Ash < Raw	
S	*	Traditional > Maassel		The direction of Maassel is rising while of traditional is falling.
Sr	–	Traditional > Maassel		
B	–	Traditional > Maassel		
Si	*		Ash < Raw	
Mn	–	Traditional > Maassel		
Ba	*	Traditional > Maassel		The direction of Maassel is rising while of traditional is falling.
Zn	*	Traditional > Maassel	Ash > Raw	
Ti	*	Traditional > Maassel	Ash < Raw	The content of the ash of Maassel tobaccos equals its raw.
Cu	–		Ash > Raw	
Pb	*			The direction of Maassel is rising while of traditional is falling.
Ni	*		Ash > Raw	The content of the ash of traditional tobaccos equals its raw.
Mo	*	Traditional > Maassel	Ash > Raw	
Se	*		Ash < Raw	
V	–	Traditional > Maassel		
Cr	*	Traditional > Maassel	Ash < Raw	The content of the ash of Maassel tobaccos equals its raw.
Ce		Traditional > Maassel	Ash < Raw	The content of the ash of Maassel tobaccos equals its raw.
Sn	–			
Co	–	Traditional > Maassel		
Sb	–			
Cd	–			
As	*		Ash > Raw	
Hg			Ash < Raw	The direction of Maassel is falling while of traditional is rising.
Total concentration	*	Traditional > Maassel	Ash > Raw	The direction of change about content of traditional is falling.

The hazard quotient or Health Risk Index is calculated from the following equation:

$$HQ=\frac{CDI}{R_{f}DO}$$

$$CDI\left(mgkg^{-1}day^{-1}\right)=\frac{CF\times IR\times EF\times ED}{BW\times AT}$$

where CDI is chronic daily intake (mg kg^−1^ day^−1^), RfDo is the oral reference dose (mg kg−1 day^−1^(, CF is the median concentration of element (mg kg^−1^), IR is the ingestion rate of compound (kg person^−1^ day^−1^), EF is exposure frequency (365 days year^−1^), and ED is the exposure duration. The higher the CDI, the higher the HQ, and the higher the HQ, the more worrying it will be. The amount of oral reference dose (R_f_DO) is determined by international institutions and its numerical value indicates the concentration of analyte that does not cause adverse effects during human life. For carcinogenic risk, the cancer slope factor (CSF) risk assessment method is used, and for the formula for calculating the cancer slope factor with 95 % confidence in increasing the risk of cancer by contact with a potential cancer agent Gene that is consumed during human life is calculated from the following equation.

CR=CancerRisk;CDI=LifetimeAverageDailyDose;CSF=CancerSlopeFactor;

CR=CDI×CFS

The amount of CDI is directly related to the amount of the hazard potential (HQ). If the HQ value for each of the potentially toxic elements is less than one, that element has no significant risk of being toxic, and ratios greater than one for HQ indicates the potential for hazard. If the result of CR is less than or equal to 1 × 6 ^− 10^ (less than one in a million people), the risk of cancer is very low and the risk of carcinogenicity can be neglected. If it is greater than 1 × 4 ^− 10^ (less than one person per ten thousand people) indicates a high risk of cancer in humans and in the range of 1 × 6 ^− 10^ to 1 × 4 ^− 10^ indicates a tolerable carcinogenic risk to humans (Tables 8 and 9).

**Table 8:** The risk ratio of elements for Apple, Blueberry, Orange, Khansar, Borazjan tobaccos.

Element	Apple	Blueberry	Orange	Khansar	Borazjan	Inhalation reference dose (mg/m3)
As	0.000108	0.000019	0.0000	0.0001	0.0001	0.00012
Cd	0.000372	0.000167	0.0016	0.0009	0.0014	0.00001
Co	0.000125	0.000392	0.0004	0.0015	0.0006	0.0000057
Cr	0.000334	0.000365	0.0003	0.0045	0.0026	0.00028
Cu	0.000571	0.000537	0.0038	0.0060	0.0060	0.004
Hg	0.000116	0.000087	0.0001	0.0001	0.0000	0.000086
Ni	0.000039	0.000537	0.0005	0.0022	0.0013	0.02
Pb	0.000013	0.000008	0.0000	0.0001	0.0001	0.0035
Zn	0.000003	0.000005	0.0000	0.0000	0.0000	0.3

**Table 9:** Cancer risk index of elements for apple, blueberry, orange, Khansar, Borazjan tobaccos.

	Carcinogenic risk (CR)					
Element	Apple	Blueberry	Orange	Khansar	Borazjan	SF
As	2.0E-03	3.4E-04	4.9E-04	1.4E-03	1.3E-03	150
Cd	2.3E-05	1.1E-05	1.0E-04	5.9E-05	8.8E-05	6.3
Co	1.2E-04	3.8E-04	3.7E-04	1.5E-03	5.9E-04	9.8
Cr	8.2E-04	9.0E-04	7.6E-04	1.1E-02	6.5E-03	41
Cu	ND	ND	ND	ND	ND	ND
Hg	ND	ND	ND	ND	ND	ND
Ni	ND	ND	ND	ND	ND	ND
Pb	1.9E-06	1.2E-06	1.4E-06	2.1E-05	1.1E-05	0.042
Zn	6.0E-04	9.4E-04	2.3E-04	2.4E-03	2.2E-03	1.5

## Conclusion

This study analyzed the metal content of two kinds of hookah tobaccos (traditional and Maassel) before and after smoking, as well as in water and smoke. It found that traditional tobaccos had more metals than Maassel tobaccos, but Maassel tobaccos had higher metal increase after smoking. Water absorbed only a small number of total metals. The metal concentrations varied depending on the type and flavor of tobaccos. Ni and Cd were the most and least abundant metals, respectively, in raw and ash. Cr was the most abundant metal in water. our methods provide valuable insights into the metal content variations in different hookah tobaccos and their behavior during smoking. However, further research is needed to fully understand the mechanisms of metal transfer during smoking.

## Acknowledgments

The authors wish to state their thanks to Islamic Azad university South Tehran Branch for supporting this work.

## CRediT authorship contribution statement

**Lida Nikbakhtan:** Data curation, Writing – original draft. **Jalal Hassan:** Methodology. **Ali Pourshaban-Shahrestani:** Writing – review & editing, Data curation. **Seyyed Hamid Ahmadi:** . **Mohammadreza Manafi:** . **Leila Torkian:** .

## Declaration of Competing Interest

The authors declare that they have no known competing financial interests or personal relationships that could have appeared to influence the work reported in this paper.

## Data Availability

Data will be made available on request.
